# NMR-Based Metabolomics Identify Metabolic Change in Spleen of Idiopathic Thrombocytopenic Purpura Patients

**DOI:** 10.3390/metabo12060565

**Published:** 2022-06-19

**Authors:** Shi Wen, Zhenzhao Wang, Jianghua Feng, Yuanyuan Yang, Xianchao Lin, Heguang Huang

**Affiliations:** 1 Department of General Surgery, Fujian Medical University Union Hospital, No. 29, Xinquan Road, Gulou District, Fuzhou 351001, China; oopsreach@126.com (S.W.); yuuuuan@163.com (Y.Y.); linxc07@163.com (X.L.); 2 Department of Electronic Science, Fujian Provincial Key Laboratory of Plasma and Magnetic Resonance, Xiamen University, No. 422, Siming South Road, Siming District, Xiamen 361005, China; hnzzxzwzz@163.com

**Keywords:** idiopathic thrombocytopenic purpura, metabolomics, biomarkers

## Abstract

Idiopathic thrombocytopenic purpura (ITP) is a common hematological disease and the abnormal platelet destruction in the spleen is a critical pathological mechanism for ITP. However, the metabolomic change in the spleen caused by ITP is still unclear. In the present study, the metabolomic information of 18 ITP and 20 normal spleen samples were detected by using ^1^H high-resolution magic angle spinning NMR spectroscopy (^1^H MAS NMR). Compared with normal spleen, the concentrations of acetate, alanine, glutamine, glycerol, isoleucine, lysine, valine, phenylalanine, leucine, and methanol in ITP spleen tissue were elevated and 3-hydroxybutyric acid, ascorbate, asparagine, ethanol, glycogen, low-density lipoprotein, malonate, *myo*-inositol, glycerophosphocholine, pyroglutamate, and taurine were decreased. Amino acids metabolic pathways, such as branched-chain amino acids pathway, were identified as the main involved pathways based on enrichment analysis. The decrease in taurine level in the spleen was the most obvious metabolic signature involving ITP with high sensitivity and specificity to distinguish the spleen of ITP from the normal (CI: 0.825–0.982). Notably, the level of taurine in the spleen was negatively correlated with the efficacy of splenectomy (*r* = 0.622, *p* = 0.006). Collectively, the data from our study revealed previously unknown ITP-related metabolomic changes in the spleen and found a potential diagnostic and efficacy-predictive biomarker for ITP treatment.

## 1. Introduction

Idiopathic thrombocytopenic purpura (ITP) is a common hematologic disorder characterized by thrombocytopenia. For most patients, bleeding is the most common clinical manifestation. The pathology is very complex, involving diverse abnormal cellular and humoral immunity. The thrombocytopenia in ITP is mainly caused by circulating platelet autoantibody-mediated destruction and production impairing [1,2,3,4]. Most platelets were destroyed by splenic macrophages in the spleen via Fcg receptor-mediated phagocytosis. Corticosteroids and intravenous immunoglobulins can reduce the elimination of platelets by regulating the immune function of macrophages.

However, some patients do not respond favorably to medical treatments. Since having a predicted 5-year hemorrhage-related mortality ranging from 2.2% to 47.8% across different age groups, refractory patients are candidates for splenectomies [5,6]. Through a splenectomy, the autoimmune antibody-induced macrophage cytotoxicity of platelets is impaired, interfering with the interactions between T cells and B cells and leading to the dysfunctional synthesis of antibodies. By far, a splenectomy remains the most effective treatment for refractory ITP patients, exhibiting the highest curative potential [6]. Unfortunately, approximately 12–14% of the patients did not benefit from a splenectomy, whereas nearly 1% and 12.9% of the patients died or developed severe surgical complications, respectively, including hemorrhage, subphrenic abscess, and thrombosis [7]. Therefore, researching the molecular pathological changes of the spleen in ITP patients may provide innovative ideas to improve the efficacy of drugs and surgical treatment. In the present study, the metabolomic changes in the spleen of ITP were detected by ^1^H high-resolution magic angle spinning NMR spectroscopy, trying to figure out the underlying pathological changes in metabolic level and the corresponding relationship with clinical outcomes.

## 2. Results

### 2.1. Metabolomic Difference between ITP and Normal Spleen

Based on durative platelet count and bleeding, thirteen, two, and three ITP patients were classified into the complete response (CR), partial response (PR), and no response (NR) groups, respectively. Histological observations of spleen tissues ensured that no spleen sample was contaminated with other primary spleen diseases (Table 1).

**Figure 1:**
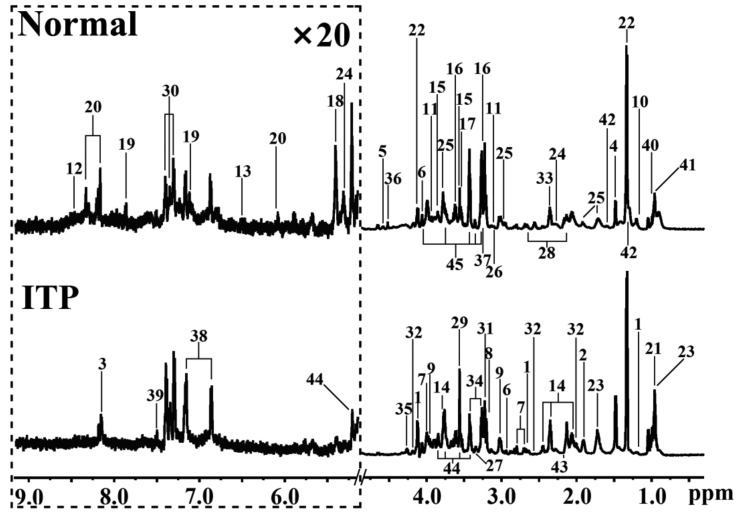
Representative HR-MAS NMR spectra of spleen tissues from the ITP patients and the normal subjects. Peaks: 1: 3-hydroxybutyrate; 2: acetate; 3: adenine; 4: alanine; 5: ascorbate; 6: asparagine; 7: aspartate; 8: choline; 9: creatine; 10: ethanol; 11: ethanolamine; 12: formate; 13: fumarate; 14: glutamate; 15: glycerol; 16: glycerophosphocholine; 17: glycine; 18: glycogen; 19: histidine; 20: inosine; 21: isoleucine; 22: lactate; 23: leucine; 24: lipid; 25: lysine; 26: malonate; 27: methanol; 28: methionine; 29: *myo*−inositol; 30: phenylalanine; 31: phosphocholine; 32: pyroglutamate; 33: pyruvate; 34: taurine; 35: threonine; 36: trigonelline; 37: trimethylamino N−oxide; 38: tyrosine; 39: uracil; 40: valine; 41: LDL; 42: VLDL; 43: glutamate; 44: alpha−glucose; 45: beta-glucose.

**Figure 2:**
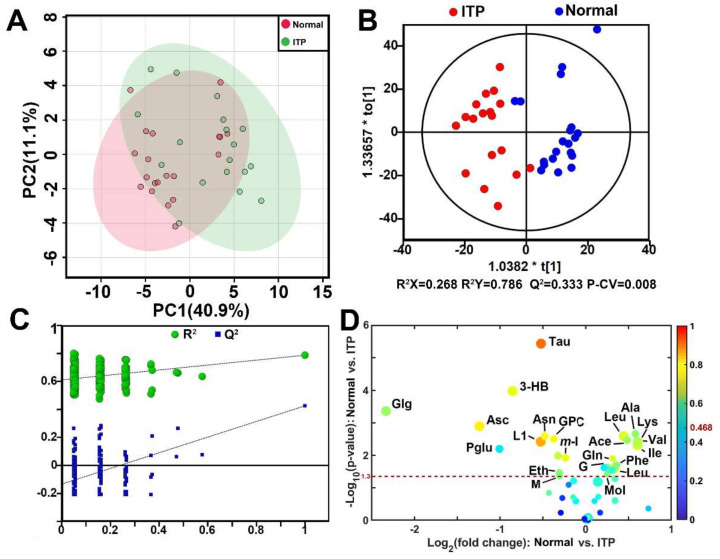
(**A**). The scores plot of principal component analysis (PCA) of ^1^H NMR spectral data of spleen derived from the ITP patients and the normal subjects. (**B**). The scores plot of orthogonal partial least squares−discriminant analysis (OPLS−DA) of ^1^H NMR spectral data. P−CV: *p*-value of CV−ANOVA analysis. (**C**). The plot of response permutation test of OPLS−DA model. (**D**). The volcano plots of discriminative metabolites between the ITP and the normal subjects. Each dot represents a metabolite. The color of dots represents the absolute value of Pcorr and the size of dot represents the VIP. The dash line represents the cut−off value for *p*-value of the discriminatory metabolites: 3−HB: 3−hydroxybutyric acid, Ace: acetate, Ala: alanine, Asc: ascobate, Asn: asparagine, Eth: ethanol; Gln: glutamine, G: glycerol, GPC: glycerophosphocholine, Glg: glycogen, Ile: Isoleucine, L1: LDL, Lys: lysine, M: malonate, Mol: methanol, m−I: *myo*−Inositol, Phe: phenylalanine, Pglu: pyroglutamate, Tau: taurine, Val: valine.

Representative ^1^H HR-MAS NMR spectra of ITP and normal spleen tissue were presented in Figure 1, and a total of 45 metabolites were identified (Table S1 in the Supplemental Materials). Through visual comparison of the NMR spectra of the ITP and normal groups, some marked metabolic differences, such as taurine and leucine, could be recognized. However, visual comparison can only provide limited metabolomic information, thus principal component analysis (PCA) was used to analyze the normalized data derived from the NMR spectra of the spleen. The corresponding PCA scores plot (Figure 2A) revealed the metabolic difference and sample separation between the ITP group and the normal group though some overlaps existed, and no distinct outliers were observed.

To highlight the metabolomic difference between ITP and normal spleen, OPLS-DA was conducted and the corresponding scores plot demonstrated a pronounced separation between ITP and normal samples (Figure 2B). The model parameters (R^2^X = 0.268, R^2^Y = 0.782, Q^2^ = 0.333) from the response permutation test (Figure 2C) and CV-ANOVA (*p* = 0.008) indicated non-overfitting and an acceptable reliability and predictability of the model. A total of 21 metabolites were identified as discriminatory metabolites between the ITP and control groups. Compared with the normal spleen, the concentrations of acetate, alanine, glutamine, glycerol, isoleucine, lysine, valine, phenylalanine, leucine, and methanol in ITP spleen tissue were elevated and 3-hydroxybutyric acid, ascorbate, asparagine, ethanol, glycogen, low-density lipoprotein, malonate, *myo*-inositol, glycerophosphocholine, pyroglutamate, and taurine were decreased (Table 2, Figure 2D). As the volcano plot demonstrated, the decrease in taurine was the most obvious metabolic signature of ITP.

**Figure 3:**
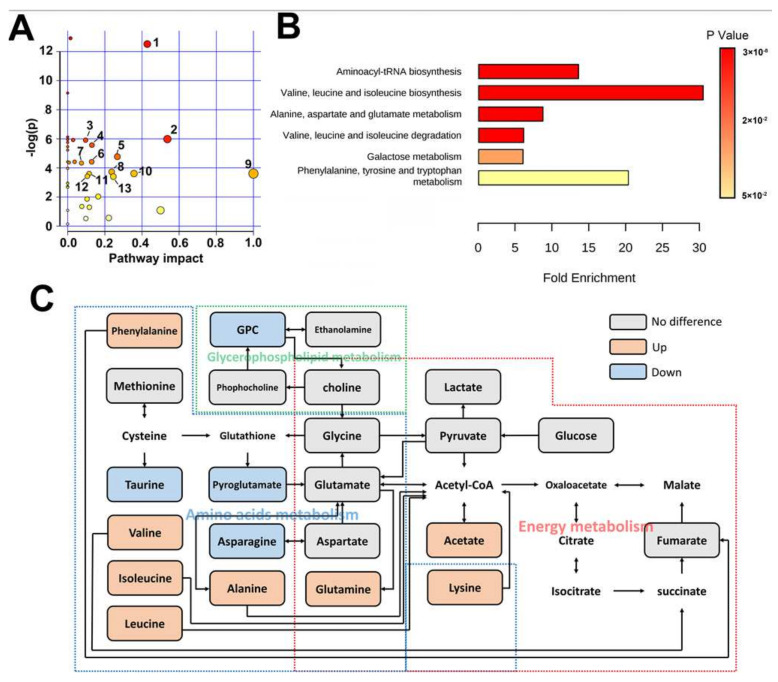
(**A**). The topological analysis of the metabolic pathway involved in metabolomic difference of spleen between ITP and normal. X axis represent the impact of metabolic pathway, and Y axis represent the −log(*p*-value) of pathway. Each dot represents a pathway, and the color of dot represent *p*-value of pathway ranging from low (Red) to high (yellow). Labeled dots represent the statistically significant metabolic pathways based on *p*-value less than 0.001 and pathway impact higher than 0.05. The numbering of Pathways: 1. Taurine and hypotaurine metabolism, 2. Alanine, aspartate and glutamate metabolism. 3. Glycerophospholipid metabolism, 4. Glycolysis/Gluconeogenesis, 5. Pyruvate metabolism, 6. Inositol phosphate metabolism, 7. Pyrimidine metabolism, 8. Glycerolipid metabolism, 9. Phenylalanine, tyrosine, and tryptophan biosynthesis, 10. Phenylalanine metabolism, 11. Glutathione metabolism, 12. Glyoxylate and dicarboxylate metabolism, 13. Glycine, serine and threonine metabolism. (**B**). The metabolic enrichment analysis of discriminative metabolites. Y axis represents the identified metabolic pathways. (**C**). The disturbed metabolic network involved in spleen of ITP. The increased, decreased, and not significantly changed levels of the metabolites in ITP groups compared with the normal are presented by orange, blue, and grey boxes. The dashed boxes with different colors indicate the different biochemical pathways. GPC: glycerophosphocholine.

Through topological and metabolite enrichment analyses, we found that the pathways of amino acid metabolism, such as branched amino acids, alanine, aspartate, phenylalanine, and tyrosine metabolism were the main metabolic change pathways involving ITP (Figure 3A,B). These discriminatory metabolites and metabolic pathways form a metabolic reprogramming network centered on amino acid metabolism, indicating a critical role of amino acid metabolism in the occurrence and development of ITP (Figure 3C).

### 2.2. Taurine Was a Potential Metabolic Marker for Diagnosis of ITP and Efficacy Predictor for Splenectomy

**Figure 4:**
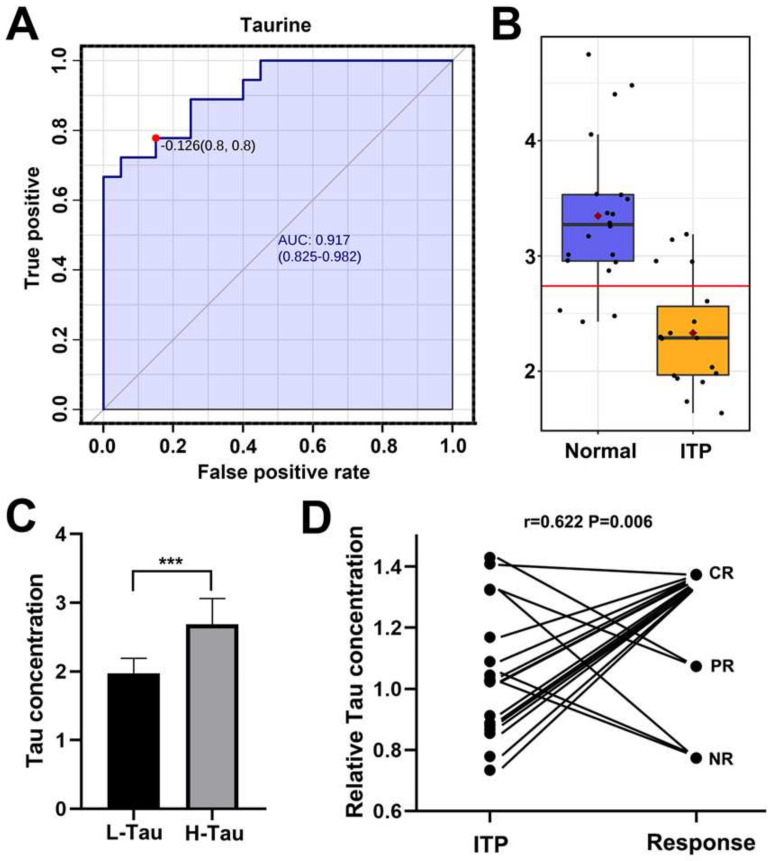
(**A**). The receiver operating characteristic (ROC) plot of taurine of spleen to distinguish immune thrombocytopenia (ITP) from the normal. (**B**). The box plot of taurine level between ITP and the normal. (**C**). The histogram of taurine level in spleen from low−taurine (L−Tau) and high−taurine (L−Tau) ITP patients (*n* = 9, respectively). *** represents *p*-value of student’s *t* test lower than 0.001. (**D**). The correlation plot between response of ITP patient to splenectomy and taurine concentration in spleen. Each black dots represent a sample. Correlation coefficient r and corresponding *p*-value were derived from *Spearman* correlation analysis; NR: no response; PR: partial response; CR: complete response.

Through receiver operating characteristic (ROC) analysis, we found the area under the curve (AUC) of taurine was 0.912 (CI: 0.825–0.982), and the decrease in spleen taurine level has a high sensitivity and specificity to distinguish ITP from the normal (Figure 4A,B). When used as a diagnostic tool to distinguish ITP from the normal, taurine could have a 0.8 sensitivity and a 0.8 specificity, indicating to be a potential marker for ITP diagnosis.

Moreover, we evaluated the underlying association between taurine concentration and the efficacy of a splenectomy. According to taurine levels, we divided ITP patients into high taurine and low taurine group (H-tau and L-tau, *n* = 9, respectively) (Figure 4C). During follow-up, the thrombocytopenia of all patients in L-tau groups (*n* = 9) was in remission without needing special treatment, while only 45% of patients in H-tau groups were in remission of thrombocytopenia (Table 1). The rate of splenectomy inefficiency in H-Tau ITP patients was significantly higher than L-Tau (*p* = 0.029). The *Spearman* correlation analysis indicated the level of taurine is negatively correlated with the response to a splenectomy (*r* = 0.622, *p* = 0.006) (Figure 4D).

## 3. Discussion

Achieving a definitive diagnosis of ITP has long been a challenge for clinicians because the specific pathogenesis of the disease is undetermined. The diagnostic principles for ITP were mainly based on clinical symptoms, platelet counts, and exclusive diagnosis of other secondary diseases [8]. Therefore, the development of a precise ITP diagnosis will be useful for clinical management. In addition, due to the severe complications that accompany splenectomy, the methods to determine preoperatively whether patients with ITP would benefit from splenectomy are also of concern to surgeons. Given that spleen is the major site of platelet destruction in ITP patients, the corresponding pathological changes in the spleen of ITP patients were obvious, including dilation and congestion of splenic sinus and macrophage infiltration. These pathological changes inevitably cause metabolic alterations in the spleen of ITP patients, which have not yet been reported. Whether these metabolic changes correlate with the responsiveness to treatment also remains unclear.

In the present study, multiple amino acids were elevated in ITP, including glutamine and branched-chain amino acids (BCAAs). Elevated amino acid levels may relate to the metabolic reprogramming of immune cells in the spleen during their activation, especially macrophages, and meet metabolic and regulatory demands upon activation of immune cells in the spleen of ITP. For immune cells, the utilization of glutamine is linked to functional activities such as cytokine production, nitric oxide production, superoxide production, and phagocytosis. Many immune cells including lymphocytes and macrophages utilize glutamine at a higher rate than glucose in immunological response [9]. Most glutamine is converted into glutamate, aspartate, and alanine to provide metabolic precursors for biosynthesis and oxidative phosphorylation, in a process called glutaminolysis. In addition, glutamine can control the proliferation of immune cells through activation of proteins such as ERK and JNK kinases, which act on transcription factors JNK and AP-1, etc., and finally leading to the transcription of cell proliferation-related genes [10]. What is more, glutamine metabolism is also involved in the polarization of macrophages to different phenotypes (M1 and M2). The deprivation of glutamine seems to decrease the M2 polarization of macrophages [11].

BCAAs, including leucine, isoleucine, and valine, are essential amino acids for immune cells which can be incorporated into proteins or oxidized into branched-chain ketoacids (BCKAs) for biosynthesis and further oxidative phosphorylation. Previous reports demonstrated that immune cells have high branched-chain amino acid transaminase (BCAT) and branched chain ketoacid dehydrogenase (BCKD) activity levels and the uptake rate of BCAAs is highly increased, especially in response to mitogens [12]. In addition to biosynthesis and oxidative phosphorylation, BCAT1-mediated BCAA catabolism controls metabolic reprogramming in activated human macrophages. In LPS-stimulated macrophages, the transamination by BCAT1 increased during early macrophage activation [13]. By inhibiting BCAT1, the oxygen consumption and glycolysis decreased, which was associated with the reduced expression level of immune responsive gene 1 (IRG1) and itaconate synthesis, resulting in decreases in itaconate, α-ketoglutarate, and 2-hydroxyglutarate levels [13,14]. In addition, BCKAs, the catabolite of BCAT1-mediated BCAA catabolism, can be excreted by tumor cells, and then be absorbed by tumor-associated macrophages (TAMs). Absorbed BCAKs can be re-animated to BCAAs and reduced the phagocytic activity of TAMs. Thus, BCAA metabolism plays a critical role in the functional modulation of immune cells, especially macrophages [15].

In addition to elevated amino acids such as glutamine and BCAAs, we found taurine is significantly decreased in the ITP spleen and, interestingly, the level of taurine seemed to be negatively correlated with the long-term response of ITP patients toward splenectomy. Taurine is a free amino acid that is abundant in the brain, heart, retina, and platelets. Previous studies have demonstrated that platelet destruction mainly occurs in the reticuloendothelial system of the spleen and liver [16]. Platelet destruction could lead to the abundant release of inner taurine. However, the variations of taurine observed in this study showed the opposite pattern, indicating that specific mechanisms associated with the immune system and inflammation may be involved [17,18]. As demonstrated in previous reports, taurine also occurs at high concentrations in phagocytes and leukocytes and accumulates in inflammatory lesions [19,20]. Taurine reacts in vivo with hypochlorous acid (HOCl)Y, a toxic product of the myeloperoxidase system, to alleviate oxidative stress [21]. The product of the reaction, taurine chloramine, can subsequently inhibit superoxide anions (O_2_^−^) and nitric oxide (NO) to provide cytoprotection. Prolonged taurine deficiency may lead to significant leukopenia and depletion of cells in the B-cell area of the spleen [22]. In addition to being cytoprotective, taurine is recently reported to antagonize the M1 polarization of macrophages by blocking the conversion of energy metabolism to glycolysis, which is required for the M1 phenotype. Thus, the decrease in taurine may result from overconsumption due to the enhancement of oxidative stress in the spleen, maintaining the homeostasis of the reticuloendothelial and lymphatic systems. Meanwhile, a decrease in taurine may promote M1 polarization and enhance the phagocytosis of macrophages in the spleen of ITP patients. The degree of taurine reduction in the spleen may represent the level of splenic platelet destruction, which may correlate with the expected efficacy of receiving a splenectomy.

So far, several studies have been conducted to identify preoperative and postoperative predictors to assess the efficacy of splenectomy for ITP patients. Age, previous response to glucocorticoids, the principal site of platelet sequestration, and response to intravenous infusion of immune globulin are reported to be preoperative predictors to estimate the outcome of splenectomy [7]. However, based on an influential meta-analysis report, most of the response predictors reported in previous studies are inconsistent and the corresponding predictive power is unreliable. In this study, we found that ITP patients with a high level of taurine have a poor sustained response to splenectomy. Detecting the taurine level of spleen tissue would help estimate the response to splenectomy postoperatively. Furthermore, the level of taurine of ITP can be detected by in vivo MRS, which can provide a useful indicator for predicting the response of ITP patients to splenectomy preoperatively, which may help identify the inappropriate candidates for splenectomy [23,24].

Other amino acids, such as lysine and phenylalanine, also influence the activation and modulation of immune cells. In addition to amino acids, the role of other discriminatory metabolites, such as 3-hydroxybutyric acid, ethanol, glycogen, and glycerophosphocholine in metabolic reprogramming in the spleen of ITP is still unclear and needs further extensive study.

In conclusion, we found the metabolomic alteration of the spleen caused by ITP and screened out discriminatory metabolites distinguishing ITP from the normal in the present study. In addition, taurine was also identified as a potential metabolic marker for the diagnosis and long-term response prediction of ITP. These findings could benefit our understanding of the pathophysiology of ITP and provide new diagnostic and therapeutic strategies.

## 4. Materials and Methods

### 4.1. Sample Collection

The study protocol was approved by the ethics committee of Fujian Medical University Union Hospital, Fuzhou, China, and was conducted in accordance with the 1964 Declaration of Helsinki and its later amendments or comparable ethical standards. Informed consent was obtained from all patients. Eighteen spleen samples were collected from ITP patients who received splenectomy in Fujian Medical University Union Hospital from September 2013 to August 2016. Before receiving surgery, these ITP patients had been treated systematically based on international consensus reports and practice guidelines for ITP management and still had refractory thrombocytopenia with a high hemorrhage risk [8,25]. Twenty normal spleen samples as control group were derived from patients with pancreatic benign lesions who received pancreatectomy and splenectomy, including cystadenoma (*n* = 18), pseudocyst (*n* = 1), and autoimmune pancreatitis (*n* = 1). All patients had no severe cardiovascular disease, diabetes, deficiency of liver and kidney functions, serious infection, and trauma.

### 4.2. Sample Preparation and ^1^H HR–MAS NMR Spectroscopy

After splenectomy, spleen was divided into 1 cm blocks, packaged in aluminum foil, frozen in liquid nitrogen, and stored at −80 °C. Prior to ^1^H high-resolution magic angle spinning (HR–MAS) NMR spectroscopy, the spleen samples (each approximately 20 mg) were shaped into a sphere and then inserted into zirconia rotors, after soaking with saline in deuterium oxide to maintain osmolality and provide a field-lock. All the ^1^H HR-MAS NMR spectra were recorded on a Bruker 600 MHz spectrometer equipped with a triple-filed resonance (^1^H/^13^C/^31^P) high-resolution MAS probe operating at a ^1^H frequency of 600.13 Hz. The tissue samples were spun at 2500 Hz at the magic angle (54.7°) and were maintained at a constant temperature of 288 K. The spectra were acquired using a water-suppressed Carr–Purcell–Meiboom–Gill (CPMG) pulse sequence (RD-90°-(τ-180°-τ)_n_-ACQ). A spin–spin relaxation delay (2nτ) of 350 ms and a 2 s recycle delay was applied for water presaturation. Typically, 128 transients were acquired with a 32 K data point for each spectrum with a spectral width of 12 ppm.

### 4.3. Data Processing

All free induction decays (FIDs) were multiplied by an exponential weighting function equivalent to line-broadening of 0.3 Hz to increase the signal-to-noise ratio. Then, all spectra were Fourier transformed and manually corrected for the phase and baseline using MestReNova (V9.0, Mestrelab Research, Santiago de Compostela, Spain). The double peak of endogenic lactate was employed as a chemical shift reference at δ1.33. The spectral regions of δ9.7–0.7 were integrally segmented into discrete regions of 0.004 ppm and then normalized to the total sum of each spectrum as a constant sum (it is 100 in this study) for multivariate statistical analysis. Resonance assignment and metabolite identification were conducted based on the literature and public databases [26,27].

### 4.4. Statistical Analysis

To extract the bioinformation contained in the NMR spectra, multivariate statistical analyses were conducted to compare the metabolomes of ITP and normal spleen by using SIMCA (Ver.14.0, Umetrics AB, Umea, Sweden). Principal component analysis (PCA) was conducted to simplify complex data into several components to reveal obvious metabolic profiles and identify possible outliers that may interfere with the validation of the analysis. To emphasize the metabolomic differences in the spleen tissues between the ITP and control samples, pairwise comparison was performed using orthogonal partial least squares discriminant analysis (OPLS-DA) with UV-scaling. This OPLS-DA model was then validated with 10-fold cross-validation and the response permutation test (permutation number = 200). The corresponding R^2^ and Q^2^ parameters represented fitness and predictive ability of the OPLS-DA model, respectively, to distinguish spleen tissues of ITP from the normal. In addition, the variable importance in projections (VIP) and correlation coefficients (r) of each metabolite obtained from OPLS-DA represent corresponding statistical contributions to the metabolic changes of ITP. The relative concentrations of metabolites, which were calculated by the integral area under the characteristic peaks of NMR spectra, were compared with Student *t*-test by using SPSS (Ver. 23.0, IBM SPSS Statistics, Armonk, NY, USA) between the ITP and normal groups. The metabolites with VIP > 1, absolute correlation coefficient |Pcorr| > 0.468 (based on *p* < 0.05 and *df* = 16) and *p*-value of Student *t*-test less than 0.05 were identified as discriminatory metabolites. A color-code volcano plot was drawn by using Matlab (Ver.2021, MathWorks, Natick, MA, US) based on VIP, r, *p* and fold change of metabolites, where each dot represented a metabolite and a warm-toned color, (e.g., red) and a large size corresponds to a significant difference, whereas a cold-toned color and a small size correspond to no significant difference.

Enrichment analysis of metabolic pathways associated with ITP was identified based on the differential metabolites by using the Kyoto Encyclopedia of Genes and Genomes (KEGG), Metabolites Biological Role (MBRole), and MetaboAnalyst 4.0 online databases (https://www.metaboanalyst.ca/, accessed on 20 September 2019) [28,29].

By using SPSS, the receiver operating characteristic (ROC) curve was used to identify the potential diagnostic ability to distinguish ITP and the normal. *Wilcoxon rank-sum* tests were employed to compare the relative concentrations of metabolites in the spleen between the normal and ITP patients. *Chi-squared* test and *Fisher’s exact* test were used to compare statistical differences in the proportion of refractory patients in different groups. *Spearman* correlation analysis was used to assess the correlation between the postoperative response of ITP patients and the concentration of taurine in the spleen.

For statistical analysis, *p* < 0.05 was considered statistically significant.

### 4.5. Follow Up

The ITP patients were followed up for over 24 months. Based on blood platelet counts and bleeding, the ITP patients were stratified into complete response (CR, durative platelet count > 100 × 10^9/^L without bleeding), partial response (durative platelet count > 30 × 10^9/^L without bleeding), and no response (NR, durative platelet count < 30 × 10^9/^L or having hemorrhage with a period of follow up) groups. This stratification was in accordance with the recommendations of the International Working Group of ITP [30].

## Figures and Tables

**Table 1:** Clinical data of ITP patients.

No.	Gender	Age	PPC ^1^	PCD ^2^	DPC ^3^	Complication	Resp.^4^
1	Female	63	161	489	>100		CR
2	Male	41	8	59	30–100		PR
3	Female	21	25	235	30–100		PR
4	Female	23	276	503	>100		CR
5	Female	28	183	157	<30	bleeding	NR
6	Male	58	171	426	>100		CR
7	Female	78	31	345	>100		CR
8	Male	22	453	463	<30	bleeding	NR
9	Female	55	108	408	>100		CR
10	Female	20	149	215	>100		CR
11	Female	21	68	990	>100		CR
12	Male	21	288	228	>100		CR
13	Male	60	49	38	<30		NR
14	Male	38	66	150	>100		CR
15	Male	25	63	87	>100		CR
16	Male	21	289	311	>100		CR
17	Male	19	157	144	>100		CR
18	Female	22	60	421	>100		CR

^1^ Preoperative platelet count (10^9^/L) ^2^ Platelet count on discharge (10^9^/L) ^3^ Durative platelet count (10^9^/L) ^4^ Response to splenectomy, CR, complete response, PR, partial response, NR, no response.

**Table 2:** The spleen discriminatory metabolites between the ITP patients and the normal.

Metabolites	ITP vs. Normal			
	Pcorr ^1^	FC ^2^	*p* ^3^	VIP ^4^
Taurine	−0.765	0.696	3.7 × 10^−6^	2.689
3-Hydroxybutyric acid	−0.603	0.553	1.1 × 10^−4^	2.213
Glycogen	−0.524	0.198	4.4 × 10^−4^	2.146
Ascorbate	−0.634	0.423	1.3 × 10^−3^	2.072
Alanine	0.511	1.497	2.2 × 10^−3^	1.811
Lysine	0.592	1.362	2.6 × 10^−3^	2.029
Asparagine	−0.623	0.717	2.6 × 10^−3^	1.907
Glycerophosphocholine	−0.614	0.772	3.2 × 10^−3^	1.886
Acetate	0.523	1.400	3.5 × 10^−3^	1.822
LDL-1	−0.703	0.695	3.8 × 10^−3^	2.011
Valine	0.548	1.525	3.9 × 10^−3^	1.948
Isoleucine	0.583	1.523	5.0 × 10^−3^	1.953
Pyroglutamate	0.594	0.497	6.4 × 10^−3^	1.782
myo-Inositol	−0.607	0.849	1.2 × 10^−2^	1.937
Glutamine	0.589	1.246	1.3 × 10^−2^	1.656
Phenylalanine	0.496	1.282	2.1 × 10^−2^	1.876
Glycerol	0.478	1.188	2.4 × 10^−2^	1.742
Leucine	0.582	1.257	2.6 × 10^−2^	2.056
Ethanol	−0.509	0.809	3.6 × 10^−2^	1.739
Methanol	0.500	1.190	3.9 × 10^−2^	1.676
Malonate	−0.491	0.810	4.4 × 10^−2^	1.654

^1^ Pcorr: correlation coefficient, positive and negative signs indicate positive and negative correlation in the concentrations, respectively. The correlation coefficients of |Pcorr| > 0.468 were used as the cutoff value for the statistical significance. ^2^ Fold change, the concentration ratio between the ITP groups and the normal. ^3^ The *p*-value of Student’s *t* test. The *p*-values less than 0.05 were used as the cutoff value for the statistical significance. ^4^ Variable importance in projection (VIP). More than 1.0 were used as the cutoff values for the statistical significance.

## Data Availability

The NMR spectral data of the spleen of ITP and the normal was uploaded as supplementary material.

## Supplementary Materials

The following supporting information can be downloaded at: https://www.mdpi.com/article/10.3390/metabo12060565/s1, Table S1: Identified metabolites from ^1^H HR-MAS spectra of spleen from the ITP patients and the normal.

## Abbreviations

3-HB	3-Hydroxybutyrate
Asc	Ascorbate
Asn	Asparagine
β-Glc	β-Glucose
CR	Complete response
CRP	C-reactive protein
Glu	Glutamate
Gln	Glutamine
GPC	Glycerophosphocholine
Glg	Glycogen
HR–MAS	High-resolution magic angle spinning
ITP	Immune thrombocytopenia
KEGG	Kyoto Encyclopedia of Genes and Genomes
MBRole	Metabolites Biological Role
m-I	Myo-inositol
NMR	Nuclear magnetic resonance
NR	No response
OPLS-DA	Orthogonal partial least squares discriminant analysis
PC	Phosphocholine
PCA	Principal component analysis
ROC	Receiver operating characteristic
PR	Partial response
Tau	Taurine
VIP	Variable importance in projection
