# Supplementary material for: NMR-Based Metabolomics Identify Metabolic Change in Spleen of Idiopathic Thrombocytopenic Purpura Patients
Source: Metabolites. 2022 Jun 19;12(6):565. doi: 10.3390/metabo12060565 (PMC9228686; doi:10.3390/metabo12060565)
Supplement: Supplementary file 1 [file metabolites-12-00565-s001.zip › metabolites-1752874-supplementary.pdf]

Table S1 Identified metabolites from ^{1}H HR-MAS spectra of spleen from the ITP patients and the normal

Metabolites	Abbr.	Chemical shift*
3-Hydroxybuyarate	3-HB	1.2d, 2.3m, 2.41m, 4.16m
Acctate	Ace	1.92s
Adenine	Ade	8.18s, 8.21s
Alanine	Ala	1.48d
Ascorbate	Asc	4.52d
Asparagine	Asn	2.86dd,2.95dd, 3.95dd
Aspartate	Asp	2.68dd, 2.82dd, 3.98dd
Choline	Ch	3.2s
Creatine	Cr	3.04s, 3.93s
Ethanol	Eth	1.19t
Ethanolamine	EA	3.13t, 3.83t
Formate	For	8.46s
Fumarate	Fum	6.52s
Glutamate	Glu	2.07m, 2.12m, 2.35m
Glutamine	Gln	2.14m, 2.45m
Glycerol	G	3.58m, 3.66m, 3.79m
Glycerophosphocholine	GPC	3.23s, 3.67m
Glycine	Gly	3.56m
Glycogen	Glg	5.42m
Histidine	His	7.1s, 7.89s
Inosine	Ino	6.1s, 8.18s, 8.38s
Isoleucine	Ile	0.94t, 1.01d
Lactate	Lac	1.33d, 4.11q
LDL-1	L1	0.89br
LDL-2	L2	1.29br
Leucine	Leu	0.96t, 1.7m
Lipid	Lipid	2.26br, 5.32br
Lysine	Lys	1.73m, 1.93m, 3.03t, 3.763t
Malonate	M	3.11s
Methanol	Mol	3.34s
Methionine	Met	2.14s, 2.16m, 2.65t
myo-Inositol	*m*-I	3.54dd, 3.62t, 4.07t
Phenylalanine	Phe	7.33d, 7.38t,7.43m
Phosphocholine	PC	3.21s
Pyroglutamate	Pglu	2.02m, 2.54m, 4.18m
Pyruvate	Py	2.373s
Taurine	Tau	3.27t,3.43t
Threonine	Threonine	4.26m
Trigonelline	TG	4.42s
Trimethylamine N-oxide	TMAO	3.29s

Tyrosine	Tyr	6.895d, 7.19d
Uracil	Ura	7.54d
Valine	Val	0.99d
alpha-Glucose	α-Glc	3.42t, 3.71t, 3.74m, 3.84m, 5.24d
beta-Glucose	β-Glc	3.26dd, 3.41t, 3.46m, 3.49t, 3.73dd, 3.9dd, 4.65d

*: s, single peak; d, double peak; dd: dual double peak; t, triple peak; m, multiple; br, broad peak
